# Abnormal spirometry 1 year after lung transplantation may identify patients at risk for chronic lung allograft dysfunction in a multicenter cohort

**DOI:** 10.1016/j.jhlto.2025.100473

**Published:** 2025-12-23

**Authors:** Alexander R. Graham, Maria V. Grau-Sepulveda, Jamie L. Todd, Megan L. Neely, Laurie D. Snyder

**Affiliations:** a Division of Pulmonary, Allergy, and Critical Care Medicine, Duke University School of Medicine, Durham, NC; b Duke Clinical Research Institute, Durham, NC; c Department of Biostatistics and Bioinformatics, Duke University School of Medicine, Durham, NC

**Keywords:** BLAD, Spiro12M, CLAD, Lung transplant, Mortality

## Abstract

**Background:**

Baseline lung allograft dysfunction (BLAD) is the failure to achieve predicted pulmonary function after lung transplantation. BLAD, as defined by abnormal spirometry at 12 months post-transplant (Spiro12M), has been associated with increased mortality and chronic lung allograft dysfunction (CLAD) in a single-center study. We sought to validate Spiro12M in a prospective multicenter study that included bilateral (BLTx) and single (SLTx) lung recipients.

**Methods:**

The cohort included first lung recipients from 5 North American transplant centers participating in the Clinical Trials in Organ Transplantation-20 study. Abnormal Spiro12M was defined as a percent predicted forced expiratory volume in 1 second (FEV1) or forced vital capacity (FVC) at 12 months post-transplant <80% predicted based on recipient demographics, or FEV1/FVC <0.7. A modified Spiro12M definition was separately analyzed whereby recipients met at least 2 criteria. Cox regression models assessed the primary endpoints of time to probable CLAD, probable CLAD composite (including CLAD-related death/retransplantation), obstructive CLAD, or graft loss (death/retransplantation).

**Results:**

517 patients met inclusion criteria, with 229 (60.4%) BLTx and 109 (79.0%) SLTx recipients having abnormal Spiro12M. Among BLTx, abnormal Spiro12M was associated with an increased risk of probable CLAD composite (HR, 1.58; 95% CI, 1.09, 2.29; *p* = 0.015), while modified Spiro12M was associated with probable CLAD (HR, 1.70; 95% CI, 1.15, 2.52; *p* = 0.008), probable CLAD composite (HR, 1.82; 95% CI, 1.25, 2.65; *p* = 0.002), and graft loss (HR, 1.68; 95% CI, 1.14, 2.48; *p* = 0.009).

**Conclusions:**

Spiro12M and modified Spiro12M are reproducible, clinically relevant definitions that can prospectively identify BLTx at risk for CLAD.

## Background

Lung transplantation is a treatment option for individuals with end-stage lung disease. Despite improvements in clinical care, long-term lung allograft survival is limited to a median of 6 years, driven mostly by the development of chronic lung allograft dysfunction (CLAD).^1^, ^2^, ^3^ While several risk factors for CLAD have been identified,^4^ treatments for CLAD are lacking. One challenge in developing new treatments is that clinical trials require multiple years of follow-up time to accrue enough CLAD events to evaluate the effects of an intervention. An earlier marker of increased CLAD or mortality could identify those at highest risk to make CLAD prevention trials more efficient and drive innovation in treatment. In the last several years, a condition termed baseline lung allograft dysfunction (BLAD) has been described, which may represent an increased risk for poor long-term outcomes.^5^, ^6^

In the broadest definition, BLAD is the failure to achieve predicted lung function after lung transplantation. However, the timing of BLAD determination, the use of the donor or the recipient predicted lung function, and the consideration of single versus bilateral lung transplants (BLTx) are still points of debate.^7^ In one of the earliest reports of BLAD, a single-center retrospective cohort study defined measurements of spirometry at 12 months post-transplant (Spiro12M) that did not meet recipient predicted norms as BLAD. Twelve months post-transplant was chosen as it reflected a timepoint when most patients had recovered to their peak post-transplant lung function.^8^ In this single-center study, abnormal Spiro12M was associated with both an increased risk of mortality as well as CLAD.^9^ We sought to evaluate this definition of BLAD in a multicenter, prospective cohort that included both BLTx and single lung transplant (SLTx) recipients as well as explore a modified definition of Spiro12M.

The Clinical Trials in Organ Transplantation-20 study (CTOT-20) was designed to identify clinical and mechanistic risk factors for CLAD. All pulmonary function tests (PFT) after transplant were collected by the data coordinating center on a monthly basis. Recipients meeting criteria for CLAD by PFTs then had a prospective CLAD adjudication done by the center to confirm there were no alternative causes identified for pulmonary function decline.^4^ Utilizing this dataset, we evaluated the incidence of BLAD, defined as abnormal Spiro12M, in BLTx recipients and tested the hypothesis that abnormal Spiro12M was associated with an increased risk of CLAD and/or graft loss after lung transplantation. This analysis was performed separately among BLTx and SLTx recipients. Our overall goal was to determine the utility of abnormal Spiro12M in identifying those at risk for CLAD or graft loss after transplant.

## Materials and methods

### Patient population

The analysis cohort was drawn from 803 first adult lung transplant recipients enrolled in the multicenter prospective observational CTOT-20 study and its extension study (CTOT-ES). Patients were transplanted between December 1, 2015, and August 31, 2018, at 5 North American centers. Recipients were followed to death, withdrawal, or study end in this observational study.^10^ Patients who died or withdrew within the first 90 days or completed <5 PFT after transplant were excluded from this analysis, as they were not considered eligible for CLAD. The study was approved by the institutional research board at Duke University (Pro00102592) and all respective participating sites. Transplant recipients were managed according to center-specific care protocols as previously reported.^4^

### Definition of abnormal spirometry at 12 months post-transplant

To determine if patients had normal or abnormal Spiro12M, the analysis cohort was limited to lung transplant recipients who survived ≥1 year post-transplant, had ≥2 post-transplant PFTs between 10 and 14 months post-transplant that were at least 3 weeks apart, and did not develop CLAD or need retransplantation before 1 year post-transplant. Spiro12M was categorized as normal or abnormal according to previously published methods^9^ using the 2 closest PFTs to 12 months at least 21 days apart. Briefly, patients were considered to have abnormal Spiro12M if either the percent predicted value of the average of forced expiratory volume in 1 second (FEV1) between the 2 PFTs was <80%, the percent predicted value of the average of forced vital capacity (FVC) was <80%, or the ratio of the average FEV1/FVC was <0.7, else the patient was considered to have normal Spiro12M. A more stringent, modified definition of abnormal Spiro12M was defined as meeting 2 or more of the above 3 criteria. Predicted values were computed using the Global Lung Initiative reference equations^11^ based on recipient demographics.

### Identification and adjudication of CLAD

Probable CLAD identification and adjudication were performed in CTOT-20 as previously described.^4^ Briefly, probable CLAD was defined as a ≥20% decline in the FEV1 as compared to the average of the 2 best post-transplant FEV1 values obtained at least 3 weeks apart.^3^ Probable CLAD events were adjudicated at each enrolling center and confirmed present if no clinical confounders were identified, with the date of CLAD onset defined as the date of the first FEV1 decline.^4^ Probable CLAD events were further characterized into an obstructive phenotype (obstructive CLAD) if there was no evidence of FVC loss as demonstrated by FVC_(CLAD)_/FVC_(Baseline)_≥0.8 and FEV1/FVC<0.7 at the time of CLAD onset.^2^

### Statistical analysis

Descriptive statistics were used to summarize cohort demographics; medians (first, third quartiles [Q1, Q3]) were reported for continuous variables, and frequency counts (percentages) were reported for categorical variables. Our primary endpoints were time to the development of probable CLAD, probable CLAD composite, obstructive CLAD, or graft loss. The probable CLAD composite outcome was defined as a composite of probable CLAD and CLAD-related death and retransplantation events, as confirmed by the site investigator, that occurred before meeting PFT criteria for CLAD.^4^ Graft loss was defined as a composite of retransplantation or death. Endpoints were landmarked at the first PFT used to assess Spiro12M. Kaplan-Meier analyses were used to describe the cumulative incidence of endpoints stratified by abnormal Spiro12M status and compared using the log-rank test.

We evaluated the association between abnormal Spiro12M and developing probable CLAD, probable CLAD composite, obstructive CLAD, or graft loss from the date of abnormal Spiro12M using Cox proportional hazard models. Univariate and multivariable Cox models were stratified by center, with our multivariable model adjusting for baseline characteristics and post-transplant events relevant to each studied outcome including age, lung allocation score (LAS), number of donor-recipient human leukocyte antigen (HLA) mismatches (binary indicator of 4-6 vs 0-3), primary lung graft dysfunction grade 3 (PGD3) within 72 hours, restrictive lung disease, and any of the following events in the first year after transplant: class I donor specific antibodies (DSA) (binary indicator of center-reported DSA vs not), class II DSAs (binary indicator of center-reported DSA vs not), organizing pneumonia or acute lung injury on biopsy, acute rejection or lymphocytic bronchiolitis, or cytomegalovirus infection (CMV) (binary indicator of CMV DNAemia as determined by CMV polymerase chain reaction test values above the limit of quantification of the assay employed at the enrolling center, or CMV pneumonitis as determined on allograft biopsy). Covariates were chosen based on biologic rationale for potential confounding or association with each endpoint and aimed to limit the total number of covariates for each outcome to 5-10 events per covariate.^12^

Analyses were performed separately in BLTx and SLTx recipient groups. Analyses were repeated for the modified definition of abnormal Spiro12M. The proportional hazards assumption was assessed by testing for interactions between covariates and log-time and evaluating log-log survival plots; no violations were found. For all statistical analyses, significance was defined as a 2-sided *p*-value of less than 0.05. There was less than 5% missing data for all relevant outcomes and covariates. SAS version 9.4 (SAS Institute, Inc., Cary, NC) was used for all analyses.

## Results

### Patient cohort and first-year characteristics

**Figure 1:**
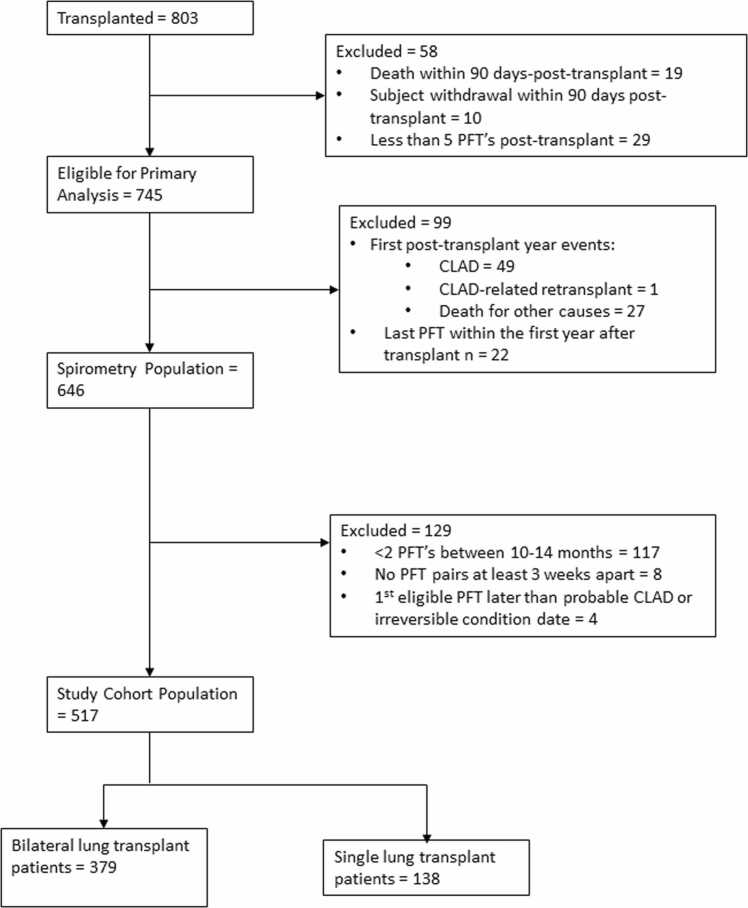
Consort diagram of study population.

803 patients underwent either first-time SLTx or BLTx at one of 5 centers in CTOT-20 during the study time frame, with 745 eligible for CLAD analysis. 517 patients met inclusion criteria for our final study cohort (Figure 1). The cohort included 379 (68.4%) BLTx and 138 (72.3%) SLTx recipients. Median [Q1, Q3] follow-up after lung transplant was 5.8 [3.3, 6.7] years. The median age at transplantation was 58 [47,63] years in BLTx and 67 [63,70] years in SLTx recipients. 160 (42%) of BLTx and 40 (29%) of SLTx recipients were female while 324 (86%) and 130 (94%) identified as White, respectively. Across BLTx and SLTx recipients, 415 (56%) were transplanted for restrictive lung disease, comprising 171 (45%) of BLTx and 115 (83%) of SLTx, respectively (Table 1).

**Table 1:** Demographics of Bilateral and Single Lung Transplant Cohorts

Variable	Level	Bilateral lung transplant			Single lung transplant		
		Overall	Normal Spiro12m	Abnormal Spiro12m	Overall	Normal Spiro12m	Abnormal Spiro12m
		(*N* = 379)	(*N* = 150)	(*N* = 229)	(*N* = 138)	(*N* = 29)	(*N* = 109)
*Sociodemographic characteristics*							
Age	Median [Q1,Q3]	58 [47,63]	58 [48,64]	58 [47,62]	67 [63,70]	68 [65,72]	67 [62,70]
Gender, *n* (%)	Female	160 (42.2)	65(43.3)	95(41.5)	40(29.0)	7(24.1)	33(30.3)
Race, *n* (%)	White	324 (85.5)	128(85.3)	196(85.6)	130(94.2)	28(96.6)	102(93.6)
	Black or African American (AA)	25 (6.6)	10(6.7)	15(6.6)	4(2.9)	1(3.4)	3(2.8)
	Other	30 (7.9)	12(8.0)	18(7.9)	4(2.9)	0(0.0)	4(3.7)
						
*Peri-transplant characteristics*							
CMV serology match, *n* (%)^a^	CMV high risk mismatch	113 (29.8)	40(26.7)	73(31.9)	31(22.5)	5(17.2)	26(23.9)
	CMV match	261 (68.9)	108(72.0)	153(66.8)	106(76.8)	23(79.3)	83(76.1)
Predicted total lung capacity, Ratio	Median [Q1, Q3]	1.0 [1.0, 1.1]	1.1 [1.0, 1.2]	1.0 [0.9, 1.1]	1.0 [0.9, 1.1]	0.9 [0.8, 1.1]	1.0 [0.9, 1.1]
Lung disease UNOS codes, *n* (%)	A	118 (31.1)	60(40.0)	58(25.3)	23(16.7)	1(3.4)	22(20.2)
	B	23 (6.1)	7(4.7)	16(7.0)	-	-	-
	C	67 (17.7)	31(20.7)	36(15.7)	-	-	-
	D	171 (45.1)	52(34.7)	119(52.0)	115(83.3)	28(96.6)	87(79.8)
End calculated LAS^b^	Median [Q1, Q3]	38.4 [34.0, 46.7]	35.8 [33.0, 44.3]	40.4 [35.0, 8.7]	37.4 [34.1, 43.4]	35.3 [33.5, 38.4]	38.0 [34.4, 44.0]
Donor-recipient HLA mismatch, *n* (%)^c^	1	4 (1.1)	0(0.0)	4(1.7)	0(0.0)	0(0.0)	0(0.0)
	2	13 (3.4)	5(3.3)	8(3.5)	3(2.2)	0(0.0)	3(2.8)
	3	52 (13.7)	19(12.7)	33(14.4)	17(12.3)	4(13.8)	13(11.9)
	4	86 (22.7)	39(26.0)	47(20.5)	47(34.1)	10(34.5)	37(33.9)
	5	135 (35.6)	54(36.0)	81(35.4)	47(34.1)	10(34.5)	37(33.9)
	6	88 (23.2)	32(21.3)	56(24.5)	24(17.4)	5(17.2)	19(17.4)
PGD Grade 3 within 72 hours, *n* (%)	Yes	76 (20.1)	16(10.7)	60(26.2)	20(14.5)	2(6.9)	18(16.5)
	No	303 (79.9)	134(89.3)	169(73.8)	118(85.5)	27(93.1)	91(83.5)

Abbreviations: CMV, cytomegalovirus; HLA, human leukocyte antigen; LAS, lung allocation score; PGD, primary graft dysfunction; Spiro12M, spirometry at 12 months post-transplant.

a CMV data is missing for 5 bilateral lung transplant and 1 single lung transplant recipients.

b LAS data is missing for 3 bilateral and 1 single lung transplant recipient (imputed to 34 to include in models).

c HLA data is missing for 1 bilateral lung transplant recipient (imputed to 4,5,6 for inclusion in models).

### Spirometry at 12 months

229 (60%) of BLTx and 109 (79%) of SLTx were found to have abnormal Spiro12M (Table 2). Notably the most common pattern was to have both FEV1 and FVC <80% predicted (54% of BLTx and 61% of SLTx). Additionally, 25% of BLTx and 9% of SLTx met abnormal Spiro12M because only one of FEV1, FVC, or FEV1/FVC components was abnormal (Table 3). After applying our modified Spiro12M criteria, 172 (45%) of BLTx and 99 (72%) SLTx recipients were found to have an abnormal modified Spiro12M.

**Table 2:** Spirometric Measurements for Abnormal Spirometry at 12 Months

	Overall	Bilateral lung transplant			Single lung transplant		
Variable	(*N* = 517)	Overall	Normal Spiro12m	Abnormal Spiro12m	Overall	Normal Spiro12m	Abnormal Spiro12m
		(*N* = 379)	(*N* = 150)	(*N* = 229)	(*N* = 138)	(*N* = 29)	(*N* = 109)
*Spirometry, Median [Q1, Q3]*							
Average FEV1	2.3 [1.8, 2.8]	2.4 [2.0, 3.0]	3.0 [2.5, 3.5]	2.2 [1.8, 2.6]	1.9 [1.5, 2.4]	2.5 [2.2, 3.0]	1.8 [1.5, 2.1]
%Predicted FEV1 [GLI]	76.8 [63.7, 90.7]	80.6 [67.2, 95.1]	98.3 [88.9, 107.5]	70.1 [61.7, 77.3]	70.3 [57.3, 80.9]	87.9 [86.6, 93.9]	62.4 [54.7, 73.2]
Average FVC	3.0 [2.4, 3.6]	3.1 [2.5, 3.8]	3.6 [3.1, 4.2]	2.8 [2.3, 3.3]	2.6 [2.1, 3.1]	3.3 [3.0, 3.6]	2.3 [2.0, 2.9]
%Predicted FVC [GLI]	78.9 [66.7, 89.7]	81.9 [69.0, 92.5]	92.7 [86.5, 101.1]	72.4 [62.0, 79.5]	71.2 [61.9, 82.1]	86.0 [82.5, 90.0]	66.6 [57.5, 75.1]
Ratio Average FEV1/Average FVC	0.8 [0.7, 0.9]	0.8 [0.7, 0.9]	0.8 [0.8, 0.9]	0.8 [0.7, 0.8]	0.8 [0.7, 0.8]	0.8 [0.7, 0.8]	0.8 [0.7, 0.8]
Ratio < 0.7, *n* (%)	98 (19.0)	63 (16.6)	0 (0.0)	63 (27.5)	35 (25.4)	0 (0.0)	35 (32.1)

Abbreviations: FEV1, forced expiratory volume in 1 second; FVC, forced vital capacity; GLI, global lung initiative.

**Table 3:** Frequencies of Abnormal Components of Spiro12M

	Bilateral lung transplant (abnormal Spiro12M *N* = 229) *n,* (%)	Single lung transplant (abnormal Spiro12M *N* = 109) *n,* (%)
Only FEV1 <80%^a^	14 (6.1)	2 (1.8)
Only FVC <80%^a^	29 (12.7)	6 (5.5)
Only FEV1/FVC <0.7^b^	14 (6.1)	2 (1.8)
FEV1 <80% and FVC <80%^a^	123 (53.7)	66 (60.6)
FEV1 <80%^a^ and FEV1/FVC <0.7^b^	24 (10.5)	11 (10.1)
FVC <80%^a^ and FEV/FVC <0.7^b^	0	0
FEV1 <80%^a^ and FVC <80%^a^ and FEV1/FVC <0.7^b^	25 (10.9)	22 (20.2)

Abbreviations: FEV1, forced expiratory volume in 1 second; FVC, forced vital capacity; Spiro12M, spirometry at 12 months post-transplant.

a Percent predicted spirometric values were calculated using global lung initiative equations based on the average of the 2 pulmonary function tests used for Spiro12M.

b FEV1/FVC ratio was calculated as a ratio of the average absolute FEV1 values to the average absolute FVC values of the 2 pulmonary function tests used for Spiro12M.

### Association between abnormal Spiro12M with CLAD or graft loss in bilateral lung recipients

**Figure 2:**
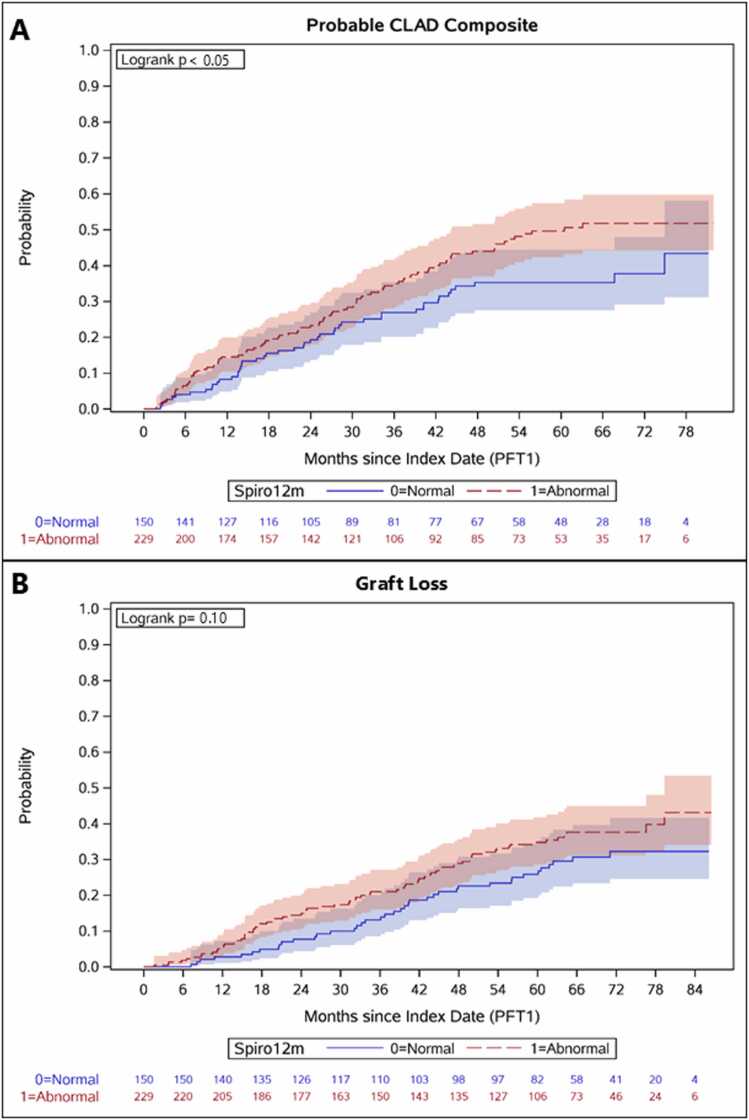
Kaplan-Meier survival curves comparing time to probable CLAD or graft loss in bilateral lung transplant recipients with abnormal Spiro12M compared to normal. **(A)** Abnormal Spiro 12M was associated with an increased risk of probable CLAD composite (Logrank *p* < 0.05). **(B)** Abnormal Spiro 12M was not associated with an increased risk of graft loss (Logrank *p* = 0.10). Abbreviations: CLAD, Chronic lung allograft dysfunction; Spiro12M, Spirometry at 12 months.

Among the BLTx cohort, abnormal Spiro12M was associated with an increased risk of the probable CLAD composite outcome (Figure 2A) (hazard ratio (HR), 1.48; 95% Confidence Interval (CI), 1.05, 2.09; *p* = 0.025) (Table 4). This association persisted after adjusting for LAS, HLA mismatch, PGD3 within 72 hours, restrictive lung disease, and the occurrence of any of the following events within the first year of transplant: class I and class II DSA’s, organizing pneumonia or acute lung injury on biopsy, acute rejection or lymphocytic bronchiolitis, and CMV (adjusted HR, 1.58; 95% CI, 1.09, 2.29; *p* = 0.015).

**Table 4:** Abnormal Spiro12M and the Relationship With CLAD, Obstructive CLAD Phenotype, and Graft Loss

Bilateral lung transplant	Unadjusted HR (95% CI), *p*-value	Unadjusted *p*-value	Adjusted HR (95% CI)	Adjusted *p*-value
Probable CLAD	1.36 (0.96, 1.94)	0.086	1.41 (0.96, 2.06)^a^	0.078
Probable CLAD-composite	1.48 (1.05, 2.09)	0.025	1.58 (1.09, 2.29)^a^	0.015
Obstructive phenotype	1.43 (0.82, 2.49)	0.203	1.53 (0.86, 2.72)^a^	0.144
Graft loss	1.30 (0.89, 1.90)	0.170	1.34 (0.91, 1.98)^b^	0.144
*Single lung transplant*				
Probable CLAD	1.45 (0.64, 3.31)	0.376	1.45 (0.61, 3.42)^a^	0.403
Probable CLAD-composite	1.62 (0.71, 3.68)	0.253	1.52 (0.64, 3.59)^a^	0.340
Obstructive phenotype^c^	0.74 (0.23, 2.42)	0.621		
Graft loss	1.62 (0.88, 2.99)	0.123	1.46 (0.76, 2.79)^d^	0.257

Abbreviations: CI, confidence interval; CLAD, chronic lung allograft dysfunction; CMV, cytomegalovirus; DSA, donor specific antigen; HLA, human leukocyte antigen; HR, hazard ratio; LAS, lung allocation score; PGD, primary graft dysfunction; Spiro12M, spirometry at 12 months post-transplant.

a Adjusted for LAS, HLA mismatch, PGD3 within 72 hours, restrictive lung disease, and any of the following events in the first year after transplant: class I and II DSA, organizing pneumonia or acute lung injury, acute rejection or lymphocytic bronchiolitis, and any CMV event.

b For age, LAS, HLA mismatch, PGD3 within 72 hours, restrictive lung disease, and any of the following events in the first year after transplant: class I and II DSA, organizing pneumonia or acute lung injury, acute rejection or lymphocytic bronchiolitis, and any CMV event.

c Multivariate analysis was not performed for obstructive phenotype in single lung transplant recipients due to insufficient events.

d Adjusted for age, LAS, HLA mismatch, PGD3 within 72 hours, restrictive lung disease, and any CMV event in the first year.

Across BLTx recipients, there was no significant association observed between abnormal Spiro12M and subsequent development of probable CLAD (Figure 2B) (HR, 1.36; 95% CI, 0.96, 1.94; *p* = 0.086), obstructive CLAD (HR, 1.43; 95% CI, 0.82, 2.49; *p* = 0.203), or graft loss (HR, 1.30; 95% CI, 0.89, 1.90; *p* = 0.170) using unadjusted traditional Cox models (Table 4). Similar results were obtained in our multivariable model. The most common cause of death for BLTx recipients with abnormal Spiro12M was progressive CLAD (Table S1).

### Association between a modified definition of abnormal Spiro12M with CLAD or graft loss in bilateral lung recipients

**Figure 3:**
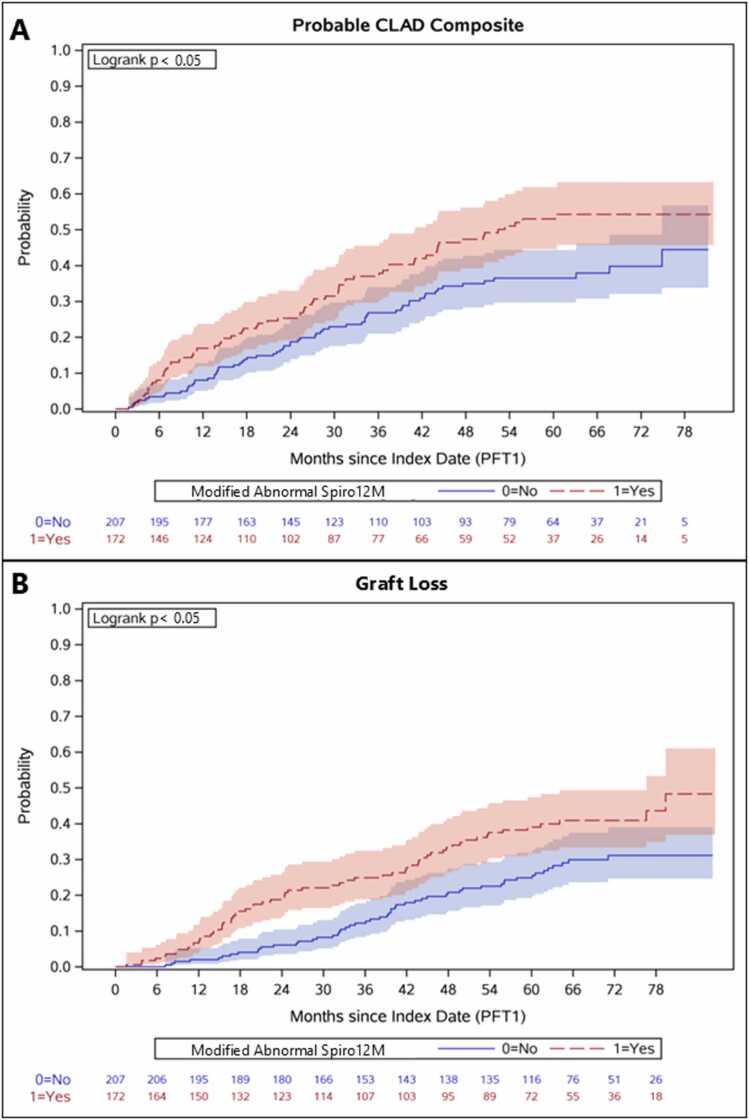
Kaplan-Meier survival curves comparing time to probable CLAD composite or graft loss in bilateral lung transplant patients with modified abnormal Spiro 12M compared to normal. **(A)** Modified abnormal Spiro 12M was associated with an increased risk of probable CLAD composite (Logrank *p* < 0.05). **(B)** Modified abnormal Spiro 12M was associated with an increased risk of graft loss (Logrank *p* < 0.05). Abbreviations: CLAD, Chronic lung allograft dysfunction: Spiro12M, Spirometry at 12 months.

An abnormal modified Spiro12M was associated with subsequent development of probable CLAD (HR, 1.57; 95% CI, 1.12, 2.21; *p* = 0.009), probable CLAD composite (Figure 3A) (HR, 1.66; 95% CI, 1.20, 2.30; *p* = 0.003), and graft loss (Figure 3B) (HR, 1.63; 95% CI, 1.13, 2.33; *p* = 0.008) in unadjusted Cox regression models (Table 5). These associations persisted in our multivariable Cox regression models for probable CLAD (adjusted HR, 1.70; 95% CI, 1.15, 2.52; *p* = 0.008), probable CLAD composite (adjusted HR, 1.82; 95% CI, 1.25, 2.65; *p* = 0.002), and graft loss (adjusted HR, 1.68; 95% CI, 1.14, 2.48; *p* = 0.009).

**Table 5:** Modified Abnormal Spiro12M and CLAD, Obstructive Phenotype, and Graft Loss

Bilateral lung transplant	Unadjusted HR (95% CI)	Unadjusted *p*-value	Adjusted HR (95% CI)	Adjusted *p*-value
Probable CLAD	1.57 (1.12, 2.21)	0.009	1.70 (1.15, 2.52)^a^	0.008
Probable CLAD-composite	1.66 (1.20, 2.30)	0.003	1.82 (1.25, 2.65)^a^	0.002
Obstructive phenotype	1.53 (0.90, 2.59)	0.116	1.75 (0.96, 3.18)^a^	0.068
Graft loss	1.63 (1.13, 2.33)	0.008	1.68 (1.14, 2.48)^b^	0.009
*Single lung transplant*				
Probable CLAD	1.70 (0.81, 3.59)	0.163	1.70 (0.76, 3.80)^a^	0.192
Probable CLAD-composite	1.86 (0.89, 3.91)	0.099	1.84 (0.83, 4.07)^a^	0.130
Obstructive phenotype^c^	0.55 (0.20, 1.46)	0.229		
Graft loss	1.38 (0.80, 2.37)	0.243	1.27 (0.70, 2.29)^d^	0.427

Abbreviations: CI, confidence interval; CLAD, chronic lung allograft dysfunction; CMV, cytomegalovirus; DSA, donor specific antigen; HLA, human leukocyte antigen; HR, hazard ratio; LAS, lung allocation score; PGD3, primary graft dysfunction grade 3; Spiro12M, spirometry at 12 months post-transplant.

a Adjusted for LAS, HLA mismatch, PGD3 within 72 hours, restrictive lung disease, and any of the following events in the first year after transplant: class I and II DSA, organizing pneumonia or acute lung injury, acute rejection or lymphocytic bronchiolitis, and any CMV event.

b Adjusted for age, LAS, HLA mismatch, PGD3 within 72 hours, restrictive lung disease, and any of the following events in the first year after transplant: class I and II DSA, organizing pneumonia or acute lung injury, acute rejection or lymphocytic bronchiolitis, and any CMV event.

c Multivariate analysis was not performed for obstructive phenotype in single lung transplant recipients due to insufficient events.

d Adjusted for age, LAS, HLA mismatch, PGD3 within 72 hours, restrictive lung disease, and any CMV event in the first year.

Abnormal modified Spiro12M was not significantly associated with an increased risk of obstructive CLAD in unadjusted (HR 1.53; 95% CI, 0.90, 2.59; *p* = 0.116) and multivariable (adjusted HR 1.75; 95% CI, 0.96, 3.18; *p* = 0.068) Cox regression models (Table 5).

### No association between abnormal Spiro12M with CLAD or graft loss in single lung recipients

In our SLTx cohort, there was no significant association between abnormal Spiro12M and the development of graft loss, probable CLAD, or probable CLAD composite (Table 4). Applying the modified Spiro12M criteria found similar results (Table 5).

## Discussion

In a multicenter cohort designed to prospectively detect the development of CLAD,^10^ our study demonstrated BLTx recipients with abnormal Spiro12M had an increased risk of a probable CLAD composite outcome (probable CLAD or investigator-confirmed CLAD-related death or retransplantation), but not probable CLAD, obstructive CLAD, or graft loss. However, BLTx recipients with abnormal lung function at 1 year by our modified Spiro12M definition have an increased risk of probable CLAD, probable CLAD composite, and graft loss. When repeated in SLTx recipients, we found no association between abnormal Spiro12M and graft loss or CLAD.

Our findings in part support a single-center, retrospective study by Paraskeva et al^9^ that initially described abnormal Spiro12M and found it was associated with both CLAD and increased mortality. However, there are several differences in our cohorts that give rise to some discrepancies. First, CTOT-20/ES had a median follow-up of 5.8 years, whereas the cohort from Paraskeva et al^9^ followed patients for a median of 6.8 years with some followed as long as 15 years after transplant, allowing more time for CLAD events to occur as reflected in their cumulative incidence of CLAD of 64% compared to 37% in our cohort. Thus, our association with only a probable CLAD composite outcome may reflect that we are capturing earlier CLAD events. This may also explain why our cohort did not demonstrate an increased risk of graft loss. Second, our cohort had a higher rate of abnormal Spiro12M (65% vs 49%), which may reflect differences in the proportions of BLTx recipients transplanted for restrictive lung disease (45% vs 11%) and obstructive lung disease (31% vs 41%) in our study compared to Paraskeva et al.^9^ Further research into BLAD may need to account for native lung disease and chest cavity size.

A specific challenge in lung transplantation is identifying the post-transplant baseline, or highest lung function, for a given patient. Post-transplant FEV1 and FVC values peak at different timepoints after lung transplantation, generally around 9 and 12 months, respectively.^8^ As a fixed timepoint measure, Spiro12M is measured when most patients may have reached their peak or baseline function. Operationalizing a fixed timepoint to be a surrogate for the baseline can address this limitation, as it can consistently be used to risk stratify and prompt intervention, allowing for use in clinical trials.

In contrast, when using a baseline of any timepoint after lung transplant to define BLAD, the variability in time limits its practical applications, as it can only be identified retrospectively.^3^ Other definitions of BLAD have used peak lung function, regardless of time from transplant, in their definition. Liu et al^6^ defined BLAD as the presence of abnormal spirometry (FEV1 or FVC <80%) at the time of post-transplant baseline, which varies between patients post-transplant and is confirmed retrospectively. In this cohort, 45% had obstructive lung disease, similar to Paraskeva, et al,^9^ however, they found that only mortality, not CLAD was associated with their definition of BLAD. Similarly, Keller et al^5^ found that BLAD after transplant was associated with mortality but not CLAD. Unlike these studies, our multicenter cohort identified an association between abnormal Spiro12M and CLAD, which may support the use of Spiro12M over other definitions of BLAD when identifying patients at risk for worse long-term outcomes post-transplant.

As we had a relatively high rate of abnormal Spiro12M using the original definition in this cohort, we explored if a more stringent definition would more closely associate with worse outcomes. Our modified definition required at least 2 PFT criteria to be abnormal in order to be classified as abnormal Spiro12M. We chose this approach, as not all patients may reach a peak in both their FEV1 and FVC by the 12-month mark, so a lower threshold of 1 criterion for abnormal Spiro12M may include patients who would otherwise be on a normal lung function trajectory. Additionally, the modified Spiro12M approach also prevented patients with normal FEV1 and FVC values from being designated as abnormal Spiro12M due solely to an FEV1/FVC ratio <0.7. We believe an abnormal modified Spiro12M may be indicative of less reserve overall, rather than identifying patients with slower rates of lung function recovery. With this modified Spiro12M definition, only 45% met the abnormal criteria, and we found this increased stringency identified BLTx recipients at risk for probable CLAD, probable CLAD composite, and graft loss, indicating that it may be a helpful surrogate to identify those at risk of poor outcomes in both clinical and research applications.

The most common cause of death adjudicated by enrolling investigators for BLTx recipients with abnormal Spiro12M was progressive CLAD, with a third occurring in patients without a prior CLAD diagnosis (TABLE S1). This mismatch highlights that prospectively adjudicating CLAD may be clinically challenging, as comorbidities and other complications may shift focus away from the diagnosis. After a patient dies, however, further examination retrospectively may make it easier to identify that CLAD had been a contributing factor, which may explain why not all patients who died of progressive CLAD had a pre-existing probable CLAD diagnosis.

SLTx recipients have only been evaluated in a retrospective single-center study of BLAD,^13^ utilizing modified criteria where normal function is defined as FEV1 and FVC >60% predicted. In these recipients, the relative contributions from native and donor lungs are uncertain, as not just lung graft volume but changes in chest wall mechanics can negatively alter the performance of the donor lung compared to predicted values.^7^ In our exploratory analysis of SLTx recipients, the higher prevalence of abnormal Spiro12M without association with CLAD or graft loss do not support utilizing the definition of abnormal Spiro12M we examined for SLTx recipients. As above, with contributions in lung volumes in SLTx being from both donor and native lungs, their PFT’s will be at higher risk of being abnormal. Further evaluation utilizing a potentially lower cutoff, such as the previously postulated value of FEV1 and/or FVC <60% may allow better characterization of the relationship between lower post-transplant lung function and post-transplant outcomes in the SLTx population.

Our study had several strengths. It utilized a multicenter cohort designed to prospectively identify risk factors for developing CLAD. Also, when evaluating for the development of CLAD, it accounted for additional CLAD-related events that would otherwise not be captured.

However, there were some limitations to this study. When detecting CLAD events, our ability to further define CLAD phenotypes was limited, as CTOT-20/ES collected spirometry data on all patients but did not uniformly collect lung volume or CT imaging data. Therefore, we were unable to further differentiate CLAD events as bronchiolitis obliterans syndrome or restrictive allograft syndrome based on current guidelines.^3^ We instead classified CLAD events into obstructive and non-obstructive events utilizing PFT data and methods that detect the preservation of FVC.^2^ Additionally, CTOT-20/ES had a limited follow-up period with a median of 5.8 years. This is less than prior single-center studies evaluating BLAD, as Paraskeva et al.^9^ followed patients a median of 6.8 years while Liu et al.^6^ followed patients a median of 6.4 years. The shorter follow up period decreases our potential to detect later CLAD events and may increase the potential to miss an association of abnormal Spiro12M and the outcomes of probable CLAD and graft loss.

An additional limitation in our study is that unlike the prior study from Paraskeva et al,^9^ our dataset did not collect information on the prevalence of potential alternative causes of reduced spirometry at 1 year post-transplant, such as diaphragm palsy, chest wall dysfunction, or anastomotic narrowing and as such we were unable to exclude them from our cohort. This could falsely elevate our observed prevalence of abnormal Spiro12M. However, even though the relationship between extrinsic causes of decreased lung function after transplant and subsequent graft loss are unknown, the lack of reserve postulated may still contribute to subsequent graft loss in these patients. Similarly, the multivariate models in our study did not account for specific donor risk factors, which may contribute to worse post-transplant outcomes. While not an extensive evaluation of all donor factors, when we evaluated for older donor age, history of smoking, or diabetes status, we found that more BLTx who had received lungs from donors aged ≥55 years went on to develop abnormal Spiro12M (Table S2). A subsequent sensitivity analysis for donors aged ≥55 years demonstrated no association with our study’s outcomes (Table S3), decreasing the likelihood that these donor factors may be confounders for our findings.

Our PFT inclusion and classification approaches carry the potential to introduce some biases in our results. First, our inclusion criteria requiring at least 2 PFT’s between 10 and 14 months increases the potential for selection bias of patients with more complicated post-transplant courses. In transplant centers where PFT’s are not routinely checked at least twice between 10 and 14 months post-transplant, a patient with 2 values could represent someone who is experiencing complications and is being more closely monitored. This could skew our cohort toward higher rates of abnormal Spiro12M. However, a descriptive comparison of patients included and excluded based on PFT availability was not notably different. Next, the use of a fixed cutoff of an FEV1/FVC ratio <0.7 increases the risk for age- and sex-related misclassification. Specifically, this approach increases the risk for inappropriately diagnosing obstructive disease in older patients and underdiagnosing obstruction in younger patient groups.^14^ In our transplanted population, there is a similar risk to identify abnormality for Spiro12M based on these criteria, however in our cohort only 6% of BLTx patients with abnormal Spiro12M patients were identified by only having an abnormal FEV1/FVC ratio <0.7, which may limit the biases from these measurements. Although 10.5% of our BLTx patients with modified abnormal Spiro12M were based off of an FEV1/FVC value <0.7, the concomitant percent predicted FEV1 <80% has a higher likelihood for identifying patients with obstruction*.*

In summary, this multi-center analysis found abnormal Spiro12M following BLTx was associated with an increased risk of experiencing probable CLAD events, including CLAD-related death and retransplantation, and when 2 or more criteria for abnormal Spiro12M were met, there was additionally an increased association with probable CLAD and graft loss. Spiro12M and the modified Spiro12M are clinically relevant definitions, as they can prospectively identify patients at risk for CLAD after lung transplantation at a fixed timepoint regardless of the trajectory of their post-transplant recovery. This information may not only help guide our thresholds for CLAD interventions but also identify patients who would enrich prospective clinical trials evaluating CLAD.

## Financial support

This work was supported by a grant from Zambon and the International Society for Heart and Lung Transplantation. CTOT-20 was supported by National Institute of Allergy and Infectious Disease award U01AI113315. CTOT-ES was supported by the Cystic Fibrosis Foundation (PALMER19AB0). AG received grant support from Zambon and the International Society for Heart and Lung Transplantation, supporting salary and project funding. LS, JT, MG, and MN have no financial conflicts of interest to disclose.

## CRediT authorship contribution statement

AG co-conceived the research study, supervised the project, oversaw data analysis, and wrote the manuscript. LS co-conceived the research study, supervised the project, oversaw data analysis, and made critical revisions to the manuscript. JT co-conceived the research study, supervised the project, oversaw data analysis, and made critical revisions to the manuscript. MG and MN were responsible for statistical analyses and made critical revisions to the manuscript. All authors discussed results and interpretations and approved the final manuscript before submission.

## Disclosure statement

The authors declare the following financial interests/personal relationships, which may be considered as potential competing interests: Alexander R. Graham reports financial support was provided by the International Society for Heart and Lung Transplantation. If there are other authors, they declare that they have no known competing financial interests or personal relationships that could have appeared to influence the work reported in this paper.

## Footnotes

Appendix A Supplementary data associated with this article can be found in the online version at doi:10.1016/j.jhlto.2025.100473.
