# Supplementary material for: Abnormal spirometry 1 year after lung transplantation may identify patients at risk for chronic lung allograft dysfunction in a multicenter cohort
Source: JHLT Open. 2025 Dec 23;11:100473. doi: 10.1016/j.jhlto.2025.100473 (PMC12857350; doi:10.1016/j.jhlto.2025.100473)
Supplement: Supplementary file 1 — Supplementary material [file mmc1.docx]

**Abnormal Spirometry One Year After Lung Transplantation May Identify Patients at Risk for Chronic Lung Allograft Dysfunction in a Multicenter Cohort**

Alexander R. Graham MD^1^, MHS, Maria V. Grau-Sepulveda MD^2^, MPH, Jamie L. Todd MD, MHS^1,2^, Megan L. Neely PhD^2,3^, Laurie D. Snyder MD, MHS^1,2^.

1. Division of Pulmonary, Allergy, and Critical Care Medicine, Duke University School of Medicine, Durham, North Carolina

2. Duke Clinical Research Institute, Durham, North Carolina

3. Department of Biostatistics and Bioinformatics, Duke University School of Medicine, Durham, North Carolina

**Corresponding Author**Alexander Graham, Duke University Medical Center, 2 Genome Ct, MSRB II, Durham, NC, 27710. Email: [alexander.graham@duke.edu](mailto:alexander.graham@duke.edu)

**Supplemental Materials**

Table of Contents

**Supplemental Tables2**

Table S1 Outcomes in bilateral lung transplant recipients 2
Table S2 Prevalence of donor risk factors in lung transplant recipients 4
Table S3 The association between receiving lung allografts from donors ≥ 55 years of age and graft loss and CLAD in bilateral lung transplant recipients 5

| **TABLE S1 Outcomes in Bilateral Lung Transplant Recipients** | | | | | | | |  |
| --- | --- | --- | --- | --- | --- | --- | --- | --- |
| **Variable** | **Level** | **Overall** | | **Normal Spiro12m** | | **Abnormal Spiro12m** | |  |
|  |  | **(N=379) (%)** | | **(N=150) (%)** | | **(N=229) (%)** | |  |
| **Outcome Numbers** |  |  |  |  |  |  |  | |
|  |  |  |  |  |  |  |  | |
| Death, *n* (%) | Yes | 103 | 27.2 | 34 | 22.7 | 69 | 30.1 | |
|  |  |  |  |  |  |  |  | |
| Retransplant, *n* (%) | Yes | 13 | 3.4 | 6 | 4.0 | 7 | 3.1 | |
|  |  |  |  |  |  |  |  | |
| Graft Loss, *n* (%) | Yes | 116 | 30.6 | 40 | 26.7 | 76 | 33.2 | |
|  |  |  |  |  |  |  |  | |
| Probable CLAD, *n* (%) | Yes | 128 | 33.8 | 46 | 30.7 | 82 | 35.8 | |
|  |  |  |  |  |  |  |  | |
| Probable CLAD Composite with CLAD-Related Deaths/Re-Transplants |  | 139 | 36.7 | 47 | 31.3 | 92 | 40.2 | |
|  |  |  |  |  |  |  |  | |
| Time from Last PFT to Graft Loss (Days) | Median [Q1, Q3] | 63 | 88.0 [46, 133] | 18 | 85.5 [40.5, 136] | 45 | 88.5 [52, 132] | |
|  |  |  |  |  |  |  |  | |
| Probable CLAD and Graft Loss Identified, *n* (%) | Yes | 59 | 50.9 | 22 | 55.0 | 37 | 48.7 | |
|  |  |  |  |  |  |  |  | |
| Time from Probable CLAD to Graft Loss | Median [Q1, Q3] | 28 | 417 [185, 705] | 10 | 531 [239, 724] | 18 | 336 [178, 577] | |
|  |  |  |  |  |  |  |  | |
| CLAD Grade among those with Probable CLAD, *n* (%) | Grade 2,3,4 [FEVPBL<=65%] | 41 | 32.0 | 16 | 34.8 | 25 | 30.5 | |
|  | Grade 0,1 [FEVPBL>65%] | 87 | 68.0 | 30 | 65.2 | 57 | 69.5 | |
|  |  |  |  |  |  |  |  | |
| CLAD Grade among those with both Probable CLAD and Graft Loss, *n* (%) | Grade 2,3,4 [FEVPBL<=65%] | 25 | 42.4 | 9 | 40.9 | 16 | 43.2 | |
|  | Grade 0,1 [FEVPBL>65%] | 34 | 57.6 | 13 | 59.1 | 21 | 56.8 | |
|  |  |  |  |  |  |  |  | |
| Death Reason (all), *n* (%) | Unknown/Missing | 22 | 21.4 | 9 | 26.5 | 13 | 18.8 | |
|  | COVID-19 | 3 | 2.9 | 2 | 5.9 | 1 | 1.4 | |
|  | Respiratory Failure | 1 | 1.0 | 0 | 0.0 | 1 | 1.4 | |
|  | Progressive CLAD | 31 | 30.1 | 8 | 23.5 | 23 | 33.3 | |
|  | Pneumonia | 7 | 6.8 | 5 | 14.7 | 2 | 2.9 | |
|  | PTLD with pulmonary involvement | 1 | 1.0 | 0 | 0.0 | 1 | 1.4 | |
|  | Cardio-Respiratory Arrest | 1 | 1.0 | 0 | 0.0 | 1 | 1.4 | |
|  | ARDS | 8 | 7.8 | 3 | 8.8 | 5 | 7.2 | |
|  | DIC | 1 | 1.0 | 0 | 0.0 | 1 | 1.4 | |
|  | Bowel Perforation | 1 | 1.0 | 1 | 2.9 | 0 | 0.0 | |
|  | Systemic Infection | 10 | 9.7 | 1 | 2.9 | 9 | 13.0 | |
|  | Pulmonary Embolism | 1 | 1.0 | 1 | 2.9 | 0 | 0.0 | |
|  | Metastatic Melanoma | 2 | 1.9 | 1 | 2.9 | 1 | 1.4 | |
|  | Esophageal Cancer | 2 | 1.9 | 0 | 0.0 | 2 | 2.9 | |
|  | Encephalopathy | 1 | 1.0 | 1 | 2.9 | 0 | 0.0 | |
|  | Cardiovascular | 9 | 8.7 | 2 | 5.9 | 7 | 10.1 | |
|  | Acute Myelogenous Leukemia | 2 | 1.9 | 0 | 0.0 | 2 | 2.9 | |
|  |  |  |  |  |  |  |  | |
| Death Reason (CLAD), *n* (%) | CLAD related death (progressive CLAD) | 31 | 30.1 | 8 | 23.5 | 23 | 33.3 | |
|  |  |  |  |  |  |  |  | |
| Probable CLAD status among those who died of progressive CLAD, *n* (%) | Yes | 21 | 67.7 | 7 | 87.5 | 14 | 60.9 | |

Abbreviations: CLAD, chronic lung allograft dysfunction; PFT, pulmonary function test; FEVPBL, Forced expiratory volume in 1 second percent baseline; PTLD, post-transplant lymphoproliferative disease; ARDS, acute respiratory distress syndrome; DIC, disseminated intravascular coagulation

| **TABLE S2 Prevalence of donor risk factors in lung transplant recipients** | | | | | | | | | | | | | |
| --- | --- | --- | --- | --- | --- | --- | --- | --- | --- | --- | --- | --- | --- |
|  |  | **Bilateral Lung Transplant** | | | | | | **Single Lung Transplant** | | | | | |
| **Variable** | **Level** | **Overall** | | **Normal Spiro12M** | | **Abnormal Spiro12M** | | **Overall** | | **Normal Spiro12M** | | **Abnormal Spiro12M** | |
|  |  | **(N=379)** | | **(N=150)** | | **(N=229)** | | **(N=138)** | | **(N=29)** | | **(N=109)** | |
| **Donor Risk Factor** |  |  |  |  |  |  |  |  |  |  |  |  |  |
|  |  |  |  |  |  |  |  |  |  |  |  |  |  |
| Donor Age $\geq$ 55 years, *n* (%) | Yes | 89 | (23.5) | 23 | (15.3) | 66 | (28.8) | 37 | (26.8) | 5 | (17.2) | 32 | (29.4) |
|  | No | 290 | (76.5) | 127 | (84.7) | 163 | (71.2) | 101 | (73.2) | 24 | (82.8) | 77 | (70.6) |
|  | Missing | 0 | (0.0) | 0 | (0.0) | 0 | (0.0) | 0 | (0.0) | 0 | (0.0) | 0 | (0.0) |
|  |  |  |  |  |  |  |  |  |  |  |  |  |  |
| Donor > 20 pack-years, *n* (%) | Yes | 45 | (11.9) | 17 | (11.3) | 28 | (12.2) | 17 | (12.3) | 4 | (13.8) | 13 | (11.9) |
|  | No | 294 | (77.6) | 115 | (76.7) | 179 | (78.2) | 111 | (80.4) | 24 | (82.8) | 87 | (79.8) |
|  | Missing | 40 | (10.6) | 18 | (12.0) | 22 | (9.6) | 10 | (7.2) | 1 | (3.4) | 9 | (8.3) |
|  |  |  |  |  |  |  |  |  |  |  |  |  |  |
| Donor history of diabetes, *n* (%) | Yes | 36 | (9.5) | 14 | (9.3) | 22 | (9.6) | 8 | (5.8) | 2 | (6.9) | 6 | (5.5) |
|  | No | 302 | (79.7) | 118 | (78.7) | 184 | (80.3) | 118 | (85.5) | 25 | (86.2) | 93 | (85.3) |
|  | Missing | 41 | (10.8) | 18 | (12.0) | 23 | (10.0) | 12 | (8.7) | 2 | (6.9) | 10 | (9.2) |
|  |  |  |  |  |  |  |  |  |  |  |  |  |  |
| **At least 1 Donor Risk Factor** | |  |  |  |  |  |  |  |  |  |  |  |  |
|  |  |  |  |  |  |  |  |  |  |  |  |  |  |
| At least 1 risk factor, *n* (%) | Yes | 144 | (38.0) | 45 | (30.0) | 99 | (43.2) | 50 | (36.2) | 9 | (31.0) | 41 | (37.6) |
|  | No | 208 | (54.9) | 93 | (62.0) | 115 | (50.2) | 79 | (57.2) | 18 | (62.1) | 61 | (56.0) |
|  | Missing | 27 | (7.1) | 12 | (8.0) | 15 | (6.6) | 9 | (6.5) | 2 | (6.9) | 7 | (6.4) |
| Abbreviations: CMV, cytomegalovirus; HLA, human leukocyte antigen; PGD, primary graft dysfunction; Spiro12M, spirometry at 12 months post-transplant | | | | | | | | | | | | | |
|  | | | | | | | | | | | | | |

| **TABLE S3. The association between receiving lung allografts from donors ≥ 55 years of age and graft loss and CLAD in bilateral lung transplant recipients** | | |
| --- | --- | --- |
| **Outcome** | ***Unadjusted HR (95% CI)*** | ***Unadjusted P-value*** |
|  |  |  |
| Probable CLAD | 1.15 (0.76, 1.74) | 0.497 |
| Probable CLAD - Composite | 1.18 (0.79, 1.75) | 0.414 |
| Obstructive Phenotype | 1.34 (0.74, 2.42) | 0.328 |
| Graft loss | 1.08 (0.71, 1.64) | 0.722 |
| Abbreviations: CLAD, chronic lung allograft dysfunction; HR, hazard ratio; CI, confidence interval. | | |
